# Comparative Description of the Expression Profile of Interferon-Stimulated Genes in Multiple Cell Lineages Targeted by HIV-1 Infection

**DOI:** 10.3389/fmicb.2019.00429

**Published:** 2019-03-12

**Authors:** Hirofumi Aso, Jumpei Ito, Yoshio Koyanagi, Kei Sato

**Affiliations:** ^1^Laboratory of Systems Virology, Institute for Frontier Life and Medical Sciences, Kyoto University, Kyoto, Japan; ^2^Graduate School of Pharmaceutical Sciences, Kyoto University, Kyoto, Japan; ^3^Division of Systems Virology, Department of Infectious Disease Control, International Research Center for Infectious Diseases, Institute of Medical Science, The University of Tokyo, Tokyo, Japan; ^4^Core Research for Evolutional Science and Technology (CREST), Japan Science and Technology Agency, Saitama, Japan

**Keywords:** interferon-stimulated gene, type I interferon, HIV-1, transcriptomics, evolution, bioinformatics

## Abstract

Immediately after viral infections, innate immune sensors recognize viruses and lead to the production of type I interferon (IFN-I). IFN-I upregulates various genes, referred to as IFN-stimulated genes (ISGs), and some ISGs inhibit viral replication. HIV-1, the causative agent of AIDS, mainly infects CD4^+^ T cells and macrophages and triggers the IFN-I-mediated signaling cascade. Certain ISGs are subsequently upregulated by IFN-I stimulus and potently suppress HIV-1 replication. HIV-1 cell biology has shed light on the molecular understanding of the IFN-I production triggered by HIV-1 infection and the antiviral roles of ISGs. However, the differences in the gene expression patterns following IFN-I stimulus among HIV-1 target cell types are poorly understood. In this study, we hypothesize that the expression profiles of ISGs are different among HIV-1 target cells and address this question by utilizing public transcriptome datasets and bioinformatic techniques. We focus on three cell types intrinsically targeted by HIV-1, including CD4^+^ T cells, monocytes, and macrophages, and comprehensively compare the expression patterns of ISGs among these cell types. Furthermore, we use the datasets of the differentially expressed genes by HIV-1 infection and the evolutionarily conserved ISGs in mammals and perform comparative transcriptome analyses. We defined 104 ‘common ISGs’ that were upregulated by IFN-I stimulus in CD4^+^ T cells, monocytes, and macrophages. The ISG expression patterns were different among these three cell types, and intriguingly, both the numbers and the magnitudes of upregulated ISGs by IFN-I stimulus were greatest in macrophages. We also found that the upregulated genes by HIV-1 infection included most ‘common ISGs.’ Moreover, we determined that the ‘common ISGs,’ particularly those with antiviral activity, were evolutionarily conserved in mammals. To our knowledge, this study is the first investigation to comprehensively describe (i) the different expression patterns of ISGs among HIV-1 target cells, (ii) the overlap in the genes modulated by IFN-I stimulus and HIV-1 infection and (iii) the evolutionary conservation in mammals of the antiviral ISGs that are expressed in HIV-1 target cells. Our results will be useful for deeply understanding the relationship of the effect of IFN-I and the modulated gene expression by HIV-1 infection.

## Introduction

Once humans are infected with pathogenic viruses, the immune systems that are composed of innate, intrinsic, and acquired immunities are evoked to combat and eliminate infected viruses. As the initial barrier against viral infections, innate immunity plays a pivotal role in triggering “danger signals” in infected individuals (Akira et al., 2006; Takeuchi and Akira, 2010).

To initiate innate immune responses, pattern recognition receptors (PRRs) such as Toll-like receptors (TLRs), retinoic acid-inducible gene-I (RIG-I; encoded by *DDX58*), melanoma differentiation-associated gene 5 (MDA5; encoded by *IFIH1*), laboratory of genetics and physiology 2 (LGP2; encoded by *DHX58*), interferon-gamma inducible protein 16 (IFI16) and cyclic GMP-AMP synthase (CGAS), recognize pathogen-specific ligands, such as double stranded RNA and CpG DNA (reviewed in Akira et al., 2006; Kawai and Akira, 2006; Takeuchi and Akira, 2010; Soper et al., 2017). The signaling pathways following to PRRs activate NF-κB and interferon regulatory factors (IRFs; IRF3 and 7) and induce the expression of type I IFNs (IFN-Is) (reviewed in Akira et al., 2006; Kawai and Akira, 2006; Takeuchi and Akira, 2010; Soper et al., 2017). Following the production of IFN-Is, including IFN-α and IFN-β, these soluble proteins bind to IFN-α/β receptors and trigger the signaling cascade mediated by Janus kinases (JAKs; JAK1 and 2), signal transducer and activator of transcriptions (STATs; STAT1 and 2), and IRF9 (reviewed in Akira et al., 2006; Kawai and Akira, 2006; Takeuchi and Akira, 2010; Soper et al., 2017). This molecular signal results in the modulation of several gene expressions. In particular, the genes upregulated by IFN-I-triggered signal are referred to as IFN-stimulated genes (ISGs), and some ISGs function as intrinsic weapons to inhibit viral infection. Namely, the PRR-mediated innate immunity triggers the antiviral actions executed by the intrinsic immunity that is composed of ISGs. As representatives of antiviral ISGs, protein kinase R (PLR; encoded by *EIF2AK2*) (Taylor et al., 1999; Balachandran et al., 2000), Viperin (encoded by *RSAD2*) (Chin and Cresswell, 2001; Wang et al., 2007; Gizzi et al., 2018) and MX dynamin-like GTPase 1 (MX1) (Melen et al., 1994; Gao et al., 2010) are widely known, and these ISGs strongly inhibit the infection of a broad range of viruses.

Human immunodeficiency virus type 1 (HIV-1), the causative agent of AIDS, targets cells that express CD4, such as CD4^+^ T cells and macrophages, for its infection. Interestingly, although both monocytes and monocyte-derived macrophages (MDMs) express CD4 and are a myeloid cell lineage, the susceptibility to HIV-1 infection is quite different between the two cell types: HIV-1 can replicate in MDMs efficiently, while HIV-1 replication in monocytes is limited at multiple steps such as post-entry and production steps (Triques and Stevenson, 2004; Srichatrapimuk and Auewarakul, 2007; Dong et al., 2009). While IFN-I has a complicated role *in vivo* (Sandler et al., 2014; Cheng et al., 2017), it suppresses HIV-1 replication at a cellular level by inducing ISGs. When humans are infected with HIV-1, at least two PRRs, CGAS (Gao et al., 2013) and IFI16 (Jakobsen et al., 2013), sense HIV-1 infection and induce IFN-I production. This HIV-1 induced IFN-I production triggers ISG expression, and certain ISGs have been thoroughly investigated as ‘restriction factors (RFs)’ or intrinsic immunity that inhibits HIV-1 replication: the apolipoprotein B mRNA editing enzyme catalytic-like 3 (APOBEC3) family [e.g., APOBEC3G (Sheehy et al., 2002)] (reviewed in Harris and Dudley, 2015), tetherin (encoded by *BST2*) (Neil et al., 2008; Van Damme et al., 2008), MX2 (Goujon et al., 2013; Kane et al., 2013), SAM domain and HD domain-containing protein 1 (SAMHD1) (Hrecka et al., 2011; Laguette et al., 2011), and tripartite motif containing 5 (TRIM5) (Stremlau et al., 2004), TRIM56 (Kane et al., 2016), IFN induced transmembrane 1-3 (IFITM1-3) (Compton et al., 2014; Foster et al., 2016), and guanylate-binding protein 2 and 5 (GBP2/5) (Krapp et al., 2016), [the detailed roles of the respective RFs are described elsewhere (Doyle et al., 2015; Harris and Dudley, 2015; Soper et al., 2017)].

The molecular mechanisms of the IFN-I production initiated by HIV-1 infection and the roles of antiviral ISGs, including RFs, in inhibiting HIV-1 replication have been thoroughly investigated (reviewed in Doyle et al., 2015). However, the similarities and differences in the expression patterns of ISGs after IFN-I stimulation among the intrinsic HIV-1 target cell types are poorly understood. In this study, we focus on the three cell types that express CD4, namely, CD4^+^ T cells, monocytes, and MDMs, and comprehensively compare their gene induction patterns following IFN-I stimulus using publicly available transcriptome data. We also compare the gene expression profiles after IFN-I stimulus to those after HIV-1 infection. We further describe the expression patterns of antiviral ISGs in each cell type and consider the evolutionary conservation of ISG expression in mammals. To our knowledge, this study is the first investigation that comprehensively compares and describes the ISG expression profile in a set of cells targeted by HIV-1 (or any other viruses).

## Materials and Methods

### RNA-Seq Data Analysis

The RNA-Seq datasets used in this study are summarized in Supplementary Tables S1, S2. Publicly available RNA-Seq data were downloaded from the NCBI SRA ^1^ and decrypted using fastq-dump command in SRA Toolkit ^2^. Sequence reads with a low sequence quality were excluded using Trimmomatic (ver. 0.36) (Bolger et al., 2014) with the options “MINLEN: 30, LEADING: 20, TRAILING: 20.” The filtered sequence reads were mapped to the human reference genome (hg38) using STAR (ver. 2.6.0c) (Dobin et al., 2013) with the gene annotation GENCODE (ver. 29) ^3^. The gene expression count matrix was generated using featureCounts (ver. 1.6.2) (Liao et al., 2014) with the GENCODE gene annotation. Genes with low expressions [<1 reads per million mapped reads (RPM) in >90% samples] were excluded from the expression count matrix. Genes that were not recorded in RefSeq (release 109) ^4^ were also excluded. The gene expression levels were normalized using voom function implemented in limma package (ver. 3.38.3) (Ritchie et al., 2015).

### Microarray Data Analysis

The microarray datasets used in this study are summarized in Supplementary Tables S3, S4. We used the raw microarray data if the raw data were available. If not, we used the preprocessed data. The raw signal density data of the microarray were normalized according to the manufacturer’s instructions (summarized in Supplementary Tables S3, S4). We only analyzed probes that were mapped to genes recorded in both GENCODE (ver. 29) (see footnote 3) and RefSeq (release 109) (see footnote 4) If several probes were available for one gene, the average value was used to represent the expression level of the gene.

### Differential Gene Expression Analysis

Prior to the analysis, the gene expression matrixes that were generated by the RNA-Seq and microarray analysis were merged, and quantile normalization was subsequently applied to the merged matrix. The normalized expression matrixes were prepared for the respective cell types. To identify ISGs in each cell type, differential gene expression analysis using limma (ver. 3.38.3) (Ritchie et al., 2015) was performed, and the fold changes and *P*-values of the genes were calculated for each cell type (referred to as the cell type-wise fold changes and *P*-values). In this analysis, information of project Ids (i.e., Ids for research projects that generated the transcriptome data) were included in the model matrix of the limma analysis to minimize the batch effects that originated from the differences in the projects. Furthermore, the differential gene expression analysis was also performed in each project, and the fold change values of the genes were calculated for each project (referred to as the project-wise fold change values). The false discovery rate (FDR) was calculated with the Benjamini–Hochberg method (Benjamini and Hochberg, 1995). A gene was regarded as an ISG in a cell type if the gene met the following criteria: (1) the cell type-wise FDR value was less than 0.05; (2) the median of the project-wise fold changes in that cell type was greater than 1.2; and (3) >70% of the project-wise fold changes in this cell type were greater than 1.

To identify differentially expressed genes (DEGs) following HIV-1 infections in CD4^+^ T cells, the differential gene expression analysis using limma was performed with the model matrix, including information of the project Ids. Additionally, the differential gene expression analysis was also performed in each project, and the fold change values of the genes were subsequently calculated for each project. A gene was regarded as a DEG if the gene met the following three criteria: (1) the overall FDR value was less than 0.05; (2) the absolute value of the median of the project-wise fold changes was greater than 1.2; and (3) the absolute values of the project-wise fold changes were greater than 1 in >70% of the projects.

### Identification of Common ISGs and Cell Type/Lineage-Specific ISGs

Common ISGs were defined as genes that were identified as ISGs in all three cell types (CD4^+^ T cells, monocytes, and MDMs). To identify cell type/lineage-specific ISGs, we first extracted genes that were identified as ISGs in a specific cell type/lineage but not in the other cell types/lineages. We subsequently compared the fold change values of these genes between the cell type/lineage in which those genes were identified as ISGs and the other cell types/lineages. If the fold change values of the genes were significantly different (FDR < 0.05 in Student’s *t*-test) between the two cell lineages, we regarded these genes as cell type/lineage-specific ISGs.

### Statistical Analysis

To determine the significant difference in the induction levels of ‘common ISGs’ (Figure 2C and Supplementary Figure S3), Welch’s *t*-test was applied.

## Results

### ISG Expression Patterns Are Different Among HIV-1 Target Cell Lineages

To investigate the effect of IFN-I stimulus on gene expressions in the intrinsic target cells for HIV-1 infection, including CD4^+^ T cells, monocytes, and MDMs, we analyzed publicly available transcriptome data of these cells treated with IFN-I. In this study, we used 14 transcriptome datasets that comprise the paired data of the cells with and without IFN-I treatment: two RNA sequencing (RNA-Seq) data and five microarray data from CD4^+^ T cells, one RNA-Seq and three microarray data from monocytes, and one RNA-Seq and two microarray data from MDMs (Table 1). To verify whether the transcriptome data obtained by RNA-Seq and microarray are usable together, we assessed the similarity of the ISG expressions measured by the two different methods. As shown in Supplementary Figure S1, the ISGs identified in RNA-Seq and microarray were highly overlapped in all three cell types although the numbers of ISGs identified in the two methods were different. Importantly, there were no biases that one method always detects higher number of ISGs compared to the other (Supplementary Figure S1). We also confirmed that the induction levels of the ISGs measured by RNA-Seq and microarray are under linear relationships in all three cell types (Supplementary Figure S2). Based on these results, we concluded that the RNA-Seq data and microarray data analyzed in this study are usable together.

**Table 1:** Transcriptome datasets of IFN-I treatment used in this study^a^.

Cell type	Project ID	Reference	Method	Cells used	IFN-I used	Concentration	Number of replicates^b^	
							
							IFN-I-treated	Untreated
CD4^+^ T cell	PRJNA258216	Linsley et al., 2014	RNA-Seq	Primary CD4^+^ T cells	IFN-β1a	60 μg/ml	3 (3)	3 (3)
CD4^+^ T cell	PRJNA308521	NA	RNA-Seq	Primary CD4^+^ T cells	IFN-α2b	NA	18 (17)	20 (19)
CD4^+^ T cell	E-GEOD-54627	Touzot et al., 2014	Microarray	Primary CD4^+^ T cells	IFN-α	10 ng/ml	24 (6)	24 (6)
CD4^+^ T cell	E-GEOD-46599	Goujon et al., 2013	Microarray	CEM	IFN-α	500–1,000 U/ml	2 (1)	2 (1)
CD4^+^ T cell	E-GEOD-46599	Goujon et al., 2013	Microarray	CEM-SS	IFN-α	500–1,000 U/ml	2 (1)	2 (1)
CD4^+^ T cell	E-GEOD-46599	Goujon et al., 2013	Microarray	Jurkat	IFN-α	500–1,000 U/ml	2 (1)	2 (1)
CD4^+^ T cell	E-GEOD-46599	Goujon et al., 2013	Microarray	Primary CD4^+^ T cells	IFN-α	500–1,000 U/ml	3 (3)	3 (3)
Monocyte	PRJNA258216	Linsley et al., 2014	RNA-Seq	Primary monocytes	IFN-β1a	60 μg/ml	3 (3)	3 (3)
Monocyte	E-GEOD-46599	Goujon et al., 2013	Microarray	THP-1	IFN-α	500–1,000 U/ml	2 (1)	2 (1)
Monocyte	E-GEOD-46599	Goujon et al., 2013	Microarray	U937	IFN-α	500–1,000 U/ml	2 (1)	2 (1)
Monocyte	E-GEOD-38351	Smiljanovic et al., 2012	Microarray	Primary monocytes	IFN-α2	100 ng/ml	7 (7)	11 (11)
MDM	PRJNA470733	NA	RNA-Seq	Primary MDMs	IFN-β	NA	10 (10)	8 (8)
MDM	E-MTAB-1437	Rasaiyaah et al., 2013	Microarray	Primary MDMs	IFN-β	10 ng/ml	2 (2)	2 (2)
MDM	E-GEOD-46599	Goujon et al., 2013	Microarray	Primary MDMs	IFN-α	500–1,000 U/ml	3 (3)	3 (3)


*NA, not applicable. ^*a*^The detail of each dataset is summarized in Supplementary Tables S1, S3. ^*b*^The numbers of technical replicates (the replicates of which source are same) and biological replicates (the replicates of which source are different) are respectively indicated. The number of biological replicates are indicated in parenthesis.*

**FIGURE 1:**
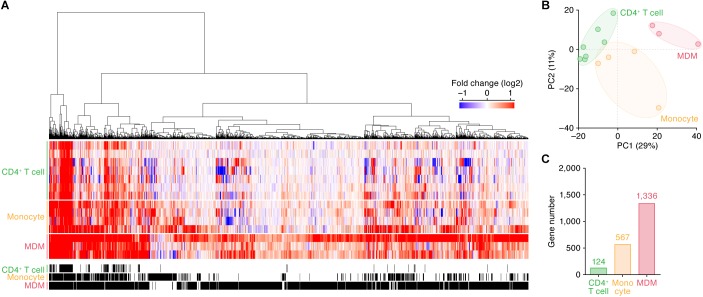
ISGs in CD4^+^ T cells, monocytes, and MDMs. **(A)** A heatmap of the ISGs in CD4^+^ T cells (seven datasets), monocytes (four datasets), and MDMs (three datasets). Color shows the fold change values of genes, and the up- and downregulated genes are indicated in red and blue, respectively. The 14 datasets used in this analysis are summarized in Table 1, and the total 1,495 ISGs are listed in Supplementary Table S5. A binary (black or white) heatmap denotes the DEGs detected in this study. **(B)** Principal component analysis of the transcriptome data based on the induction levels of ISGs upon IFN-I stimulus. PC, principal component. The result from a respective dataset is indicated by a dot. **(C)** The number of ISGs. Each number on the bar graph indicates the gene number.

We subsequently identified the ISGs in these cell types. As shown in Figure 1A, 124, 567, and 1,336 genes were upregulated by IFN-I treatment in CD4^+^ T cells, monocytes and MDMs, respectively (the total 1,495 ISGs are listed in Supplementary Table S5). In principal component (PC) analysis based on the induction levels of ISGs, the transcriptome data were separated according to their cell types (Figure 1B). These results indicate that (i) each cell type has a common pattern of ISG inductions although distinct states of cells (e.g., primary or immortalized cells) are included in the dataset, and that (ii) the IFN-I induced gene expression patterns are different among the three cell types. Moreover, the number of ISGs in CD4^+^ T cells is relatively low, while higher numbers of ISGs were detected in monocytes and MDMs (Figure 1C).

### IFN-I Strongly Affects the Gene Expression Pattern in MDMs

**FIGURE 2:**
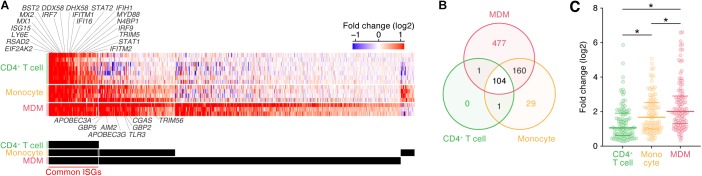
Classification of ISGs based on cell type/lineage specificity. **(A)** A heatmap of ‘common ISGs’ and cell type/lineage-specific ISGs. Color shows the fold change values of genes, and the up- and downregulated genes are indicated in red and blue, respectively. Representative 20 common ISGs and eight myeloid-specific ISGs are indicated above and below the heatmap. The total 772 ISGs and 104 ‘common ISGs’ are indicated in Supplementary Table S5. A binary (black or white) heatmap denotes the ISGs detected in the indicated cell category. **(B)** A Venn diagram of the ISGs in the three cell types. **(C)** Induction level of the 104 ‘common ISG’ expression following IFN-I treatment in the three cell types. Each dot indicates the fold change value of an ISG expression following IFN-I treatment. Horizontal lines indicate the quantiles. Asterisks indicate *P* < 0.005 by Welch’s *t*-test. Also refer to Supplementary Figure S3.

As described above, the induction patterns of genes following IFN-I stimulus were different among the three cell types (Figure 1B). To further illuminate the commonalities and differences of the ISG induction among the cell types, we classified ISGs according to the cell type specificities of their inductions following IFN-I stimuli (Figure 2A; the 772 ISGs are indicated in Supplementary Table S5). We defined ‘common ISGs,’ a set of genes that were regarded as ISGs in all three cell types. We also defined cell type- or lineage-specific ISGs, a set of genes whose expressions were induced by IFN-I stimuli in a specific cell type or lineage but not in the other cell types/lineages (see Materials and Methods). As shown in Figure 2B, 104 ‘common ISGs’ were detected in the three cell types, while the ISGs specific for CD4^+^ T cells were not detected. Moreover, 29 and 477 ISGs were specific for monocytes and MDMs, respectively, and 160 ISGs were specific for myeloid cell lineage (monocytes and MDMs) (Figure 2B). When we further examine the 104 ‘common ISGs’ (indicated in Supplementary Table S5), general antiviral genes, such as *RSAD2* (encoding Viperin), *EIF2AK2* (encoding PKR), and *MX1*, and a recently discovered ISG that potently promotes viral replication, *LY6E* (Yu et al., 2017; Mar et al., 2018), were classified as ‘common ISGs’ (Figure 2A). Additionally, well-known anti-HIV-1 RFs, such as *ISG15* (Okumura et al., 2006), *MX2, IFITM1/2, BST2* (encoding tetherin), and *TRIM5*, were ‘common ISGs’ (Figure 2A). Of the genes associated with innate immune signaling pathways, certain genes that play pivotal roles in the IFN-I induced pathway, such as *STAT1/2* and *IRF7/9* (reviewed in Soper et al., 2017), and several genes associated with the pathogen sensing pathway, such as *DDX58* (encoding RIG-I), *IFIH1* (encoding MDA5), *DHX58* (encoding LGP2), *IFI6*, and *MYD88* (reviewed in Kawai and Akira, 2006), were classified as ‘common ISGs’ (Figure 2A). On the other hand, five anti-HIV-1 RFs including *APOBEC3A, APOBEC3G, GBP2/5*, and *TRIM56* and three immunological genes including *TLR3, CGAS*, and *AIM2*, which comprise a major component that triggers inflammasome (Hornung et al., 2009), were specific ISGs for myeloid cells (Figure 2A). Previous studies have reported that *DDX58* (encoding RIG-I) and *IFIH1* (encoding MDA5) are ubiquitously expressed and work as the sensors for viral double stranded RNA in various cell lineages (reviewed in Barral et al., 2009), while CGAS plays a pivotal role in sensing HIV-1 infection in myeloid cells by detecting reverse transcribed viral DNA (Ma et al., 2015). Therefore, our results are reasonable and consistent with previous findings. Furthermore, when we focused on the induction levels of ISGs, we found that the fold changes in the expression levels of the 104 ‘common ISGs’ in MDMs were statistically greater than those in CD4^+^ T cells and monocytes (Figure 2C; the results from respective datasets are summarized in Supplementary Figure S3). Taken together, our findings suggest that both the number of ISGs and their induction levels in MDMs following IFN-I stimulus are relatively greater than those in the other two cell types.

### HIV-1 Infection Induces the Expression of the ‘Common ISGs’ With Antiviral Ability

**FIGURE 3:**
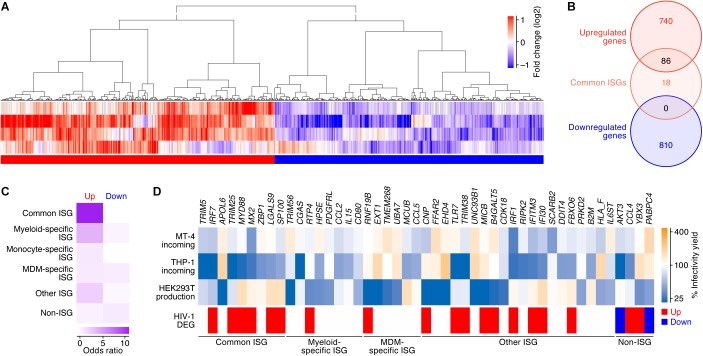
DEGs following HIV-1 infection in CD4^+^ T cells. **(A)** A heatmap of the DEGs following HIV-1 infection in CD4^+^ T cells (four datasets). Color shows the fold change values of genes, and the up- and downregulated genes are indicated in red and blue, respectively. A binary (red or blue) heatmap denotes the up- and down-regulated DEGs detected in the dataset of the HIV-1 infected CD4^+^ T cells. The four datasets used in this analysis are summarized in Table 2, and the 826 upregulated and 810 downregulated genes are, respectively, listed in Supplementary Table S6. **(B)** A Venn diagram of the 104 ‘common’ ISGs defined in this study (Figure 2B) and the DEGs by HIV-1 infection. **(C)** A heatmap showing the degree of the overlap of genes between DEGs following HIV-1 infection and each category of ISGs (Figure 2). Color shows the odds ratio of the overlap between the two gene sets. **(D)** A heatmap showing the anti-HIV-1 activity of ISGs. Upper panel shows the anti-HIV-1 activity measured by the previous study (Kane et al., 2016). “MT-4 incoming” and “THP-1 incoming” indicate the degree of the inhibition of HIV-1 infection at the early step of retroviral life cycle in each cell type, respectively. “HEK293T production” indicates the degree of the inhibition of HIV-1 production in HEK293T cells. The genes that suppressed viral infectivity/yield at the level of <50% compared to the mock-transfected cells in ≥1 dataset are shown. Lower panel shows the annotation of the genes according to our classifications of ISGs and the DEGs following HIV-1 infection.

We next examined the similarities and differences in the gene expression patterns following IFN-I stimulus and HIV-1 infection. To address the effect of HIV-1 infection on gene expression, we used the four transcriptome datasets obtained from different conditions: CD4^+^ T cells isolated from infected individuals (Sedaghat et al., 2008) and infected humanized mice (Yamada et al., 2018), and MT-4 cells and the primary CD4^+^ T cells and infected with HIV-1 in *in vitro* cultures (Lichinchi et al., 2016) (Table 2). As shown in Figure 3A, 826 and 810 DEGs were detected as the up- and downregulated genes in HIV-1 infected CD4^+^ T cells (the 826 upregulated DEGs and 810 downregulated DEGs are listed in Supplementary Table S6). When compared to the ‘common ISGs’ defined in Figure 2, 86 out of the 104 ‘common ISGs’ (82.7%) were upregulated in HIV-1 infected CD4^+^ T cells (Figure 3C), and notably, certain ‘common ISGs’ of interest (indicated in Figure 2A) were upregulated by HIV-1 infection (Supplementary Table S6). On the other hand, any ‘common ISGs’ were not downregulated by HIV-1 infection (Figure 3B). Moreover, a statistical analysis indicated that ISGs, particularly the ‘common ISGs,’ were preferentially upregulated by HIV-1 infection (Figure 3C; odds ratio = 12.97, *P* = 2.0 × 10^-51^ by Fisher’s exact test). Taken together, these findings suggest that HIV-1 infection robustly induces the ‘common ISG’ expression.

**Table 2:** Transcriptome datasets of HIV-1 infection used in this study^a^.

Cell type	Project ID	Reference	Method	Cells used	HIV-1 strain	Number of replicates^b^	
						
						HIV-1- infected	Uninfected
CD4^+^ T cell	E-GEOD-9927	Sedaghat et al., 2008	Microarray	CD4^+^ T cells from individuals	NA	11 (11)	9 (9)
CD4^+^ T cell	PRJNA374028	Yamada et al., 2018	RNA-Seq	CD4^+^ T cells from humanized mice	REJO	2 (2)	2 (2)
CD4^+^ T cell	PRJNA277687	NA	RNA-Seq	Primary CD4^+^ T cells	89.6	3 (3)	2 (2)
CD4^+^ T cell	PRJNA298743	Lichinchi et al., 2016	RNA-Seq	MT-4	LAI	2 (1)	2 (1)


*NA, not applicable. ^*a*^ The detail of each dataset is summarized in Supplementary Tables S2, S4. ^*b*^The numbers of technical replicates (the replicates of which source are same) and biological replicates (the replicates of which source are different) are, respectively, indicated. The number of biological replicates are indicated in parenthesis*.

We subsequently assessed whether the DEGs by HIV-1 infection and/or the 104 ‘common ISGs’ possess the ability to inhibit HIV-1 replication. Regarding this issue, Kane et al. (2016) have recently performed a comprehensive ISG screening against HIV-1 infection in MT-4 cells (a CD4^+^ T cell line) and THP-1 cells (a myeloid cell line) and HIV-1 production from HEK293T cells. We used this dataset and extracted 43 ISGs with anti-HIV-1 activity on either viral infection (in MT-4 cells or THP-1 cells) or viral production from HEK293T cells. Compared to our findings, *IRF7, TRIM25*, and *MX2* were the ‘common ISGs’ that exhibited anti-HIV-1 effects in MT-4 cells and were upregulated by HIV-1 infection (Figure 3D). Moreover, certain ‘common ISGs’ that were upregulated by HIV-1 infection (*IRF7, TRIM25, MYD88, MX2, LGALS9*, and *SP100*) exhibited a strong anti-HIV-1 effect in THP-1 cells (Figure 3D). Taken together, these results suggest that certain ‘common ISGs’ are upregulated by HIV-1 infection and exhibit an anti-HIV-1 effect.

### ‘Common ISGs’ Are Evolutionarily Conserved in Mammals With Antiviral Ability

**FIGURE 4:**
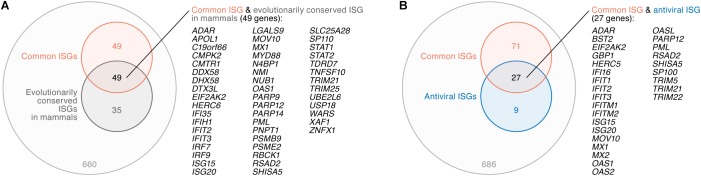
Relationship between ‘common ISGs’ and ‘evolutionary core ISGs in mammals.’ **(A)** A Venn diagram of ‘common ISGs’ and ‘evolutionary core ISGs in mammals.’ **(B)** A Venn diagram of ‘common ISGs’ and antiviral ISGs. Both ‘evolutionary core ISGs in mammals’ **(A)** and antiviral ISGs **(B)** were defined in a previous paper (Shaw et al., 2017). The universal set of panels **(A,B)** is the 793 ISGs (refer to text). The genes overlapped in both groups are listed on the right of the respective Venn diagrams.

A previous investigation by Shaw et al. (2017) addressed the evolution of ISGs in mammals including human, rat, cow, sheep, pig, horse, dog, and bats, and has indicated the presence of ‘evolutionary core ISGs’ whose sensitivity to IFN-I are evolutionarily conserved among mammals. To address whether the ‘common ISGs’ in CD4^+^ T cells, monocytes, and MDMs defined in this study (Figure 2) are evolutionarily conserved in mammals, we compared our results to the findings by Shaw et al. (2017). We detected 793 genes that were defined as ISGs in both our data and the human data obtained in the previous study (Shaw et al., 2017), and 84 out of the 793 genes were classified as ‘evolutionary core ISGs’ in the previous study (Shaw et al., 2017). In the 793 ISGs, 98 out of the 104 ‘common ISGs’ were included (also refer to Supplementary Table S5). Intriguingly, 49 out of the 98 ‘common ISGs’ (50.0%) were overlapped with ‘evolutionary core ISGs,’ while only 35 out of the 695 ‘non-common ISGs’ in this study (5.0%) were classified as ‘evolutionary core ISGs’ (Figure 4A). In particular, three major PRR-encoding ‘common ISGs,’ *DDX58* (encoding RIG-I), *DHX58* (encoding LGP2) and *IFIH1* (encoding MDA5), and general antiviral ‘common ISGs,’ such as *EIF2AK2* (encoding PKR), *MX1* and *RSAD2* (encoding Viperin), were evolutionarily conserved in mammals (Figure 4A). As this overlap of ‘common ISGs’ and ‘evolutionary core ISGs’ is statistically significant (odds ratio = 18.7, *P* = 2.9 × 10^-28^ by Fisher’s exact test), these results suggest that the ‘common ISGs’ are highly conserved and evolutionarily play a crucial role in antiviral action in mammals.

Moreover, Shaw et al. (2017) indicated that antiviral ISGs tend to be evolutionarily conserved. To assess whether the ‘common ISGs’ defined in this study (Figure 2) possess antiviral ability, we compared our results to the previous report (Shaw et al., 2017). In the 793 ISGs, 36 genes were defined as antiviral ISGs by Shaw et al. (2017). Compared to our results, 27 out of the 36 antiviral ISGs (75.0%), such as *BST2* (encoding tetherin), *EIF2AK2* (encoding PKR), *MX1*, and *MX2*, were included in the ‘common ISGs’ defined in this study (Figure 4B). As ‘common ISGs’ were highly overlapped with antiviral ISGs with statistical significance (odds ratio = 28.7, *P* = 3.7 × 10^-29^ by Fisher’s exact test), our findings suggest that the ‘common ISGs’ in CD4^+^ T cells, monocytes and MDMs potently exhibit an antiviral effect.

## Discussion

In this study, we defined the 104 ‘common ISGs’ that were commonly upregulated by IFN-I treatment in CD4^+^ T cells, monocytes, and MDMs (Figure 1, 2). We also detected the DEGs by HIV-1 infection and considered the association with IFN-I stimulus and anti-HIV-1 activity (Figure 3). Moreover, we showed that the ‘common ISGs,’ particularly those with antiviral ability, were evolutionarily conserved in mammals (Figure 4). To our knowledge, this study is the first investigation to comprehensively address (i) the similarities and differences of ISG expression among potent HIV-1 target cells; (ii) the association between ISG expression and the DEGs by HIV-1 infection; and (iii) the evolutionary conservation in mammals and antiviral effect of the ISGs commonly expressed in HIV-1 target cells by IFN-I stimulus.

It is well known that myeloid cells, particularly MDMs/macrophages, are a cell lineage specialized for inducing innate immune responses and are one of the major IFN-I producers by sensing pathogens (reviewed in Akira et al., 2006; Kawai and Akira, 2006; Takeuchi and Akira, 2010; Soper et al., 2017). In addition to these observations, we found that the sensitivity of MDMs to IFN-I stimulus, namely, the numbers of both ISGs (1,336 genes; Figure 1C) and specific ISGs (477 genes; Figure 2B), and the magnitude of the induction level of 104 ‘common ISGs’ (Figure 2C) by IFN-I treatment were greatest in MDMs compared to CD4^+^ T cells and monocytes. These results may explain the reason why IFN-I treatment exhibits higher antiviral effects on MDMs than CD4^+^ T cells (Goujon and Malim, 2010). Moreover, we found that the induction levels of 104 ‘common ISGs’ in monocytes were statistically higher than those in CD4^+^ T cells (Figure 2C), and 29 ISGs were specific for monocytes (Figure 2B). It is possible that these monocyte-specific ISGs are associated with the resistance of monocytes to HIV-1 infection (Triques and Stevenson, 2004; Srichatrapimuk and Auewarakul, 2007; Dong et al., 2009).

ISG expression analyses indicated a different expression pattern of two potent HIV-1 sensors, *IFI16* and *CGAS*: *IFI16* was commonly induced in the three cell types following IFN-I stimulus, while *CGAS* was specifically induced in myeloid cells (Figure 2A). These observations are consistent with previous reports (Gao et al., 2013; Jakobsen et al., 2013) and suggest that CGAS is a specific PRR in myeloid cells. Moreover, following HIV-1 infection in CD4^+^ T cells, various ISGs, including ‘common ISGs,’ were clearly upregulated (Figure 3). These findings suggest that IFI16 plays a crucial role in sensing HIV-1 infection in CD4^+^ T cells, resulting in IFN-I production and ISG expression. In addition to the upregulated genes, we detected 810 downregulated genes by HIV-1 infection (Figure 2A), which indicates that HIV-1 infection modulates various gene expressions other than the upregulation of ISGs. Interestingly, *PABPC4*, a downregulated gene by HIV-1 infection, harbors the ability to suppress HIV-1 production (Figure 3D) (Kane et al., 2016). PABPC4 is a mRNA poly(A) binding protein (Burgess et al., 2011), and a previous study reports that PABPC4 forms a ribonucleoprotein complex with some HIV-1 encoding proteins, such as Gag and Nef (Milev et al., 2012). Although how PABPC4 exhibits an antiviral effect and how HIV-1 infection downregulates *PABPC4* remain unclear, these findings suggest that HIV-1 may counteract PABPC4-mediated antiviral action by downregulating this gene through the formation of the ribonucleoprotein complex of viral proteins and PABPC4.

When we consider the evolutionary conservation of ‘common ISGs,’ 10 genes (*ADAR, EIF2AK2, ISG15, ISG20, MOV10, MX1, OAS1, PML, RSAD2, SHISA5*, and *TRIM21*) were evolutionarily conserved in mammals and possess antiviral activity (Figure 4). In addition to the three well-known antiviral genes (*EIF2AK2, MX1*, and *RSAD2*), *MOV10* is included in this category. Interestingly, it has been reported that *MOV10* possesses inhibitory activity against HIV-1 (Burdick et al., 2010; Furtak et al., 2010; Abudu et al., 2012) as well as endogenous retroviruses (Goodier et al., 2012; Lu et al., 2012). As *MOV10* is an evolutionarily conserved antiviral ISG, this gene may be closely associated with mammalian evolution, including retroviral endogenization and the long-lasting battle with exogenous pathogenic retroviruses, including HIV-1.

Using public transcriptome datasets, we investigated the expression patterns of ISGs among HIV-1 target cells, CD4^+^ T cells, monocytes, and MDMs and showed that the expression patterns of ISGs and their induction levels following IFN-I stimulus were different among these three cell types. Future investigations using datasets from various cell types, such as mucosal cells, hepatocytes, and neuronal cells, that can be the major targets for viral infections, will provide further understanding of the cell-type specific gene regulation triggered by IFN-I. However, as shown in Table 1, the concentrations and subtypes of IFN-I used in respective datasets are different. Moreover, although the expression level of ISGs is kinetically different (Bolen et al., 2014; Jilg et al., 2014), the information on the duration for IFN-I treatment was publicly unavailable. These issues suggest that our investigation may underestimate the effect of IFN-I treatment. Nevertheless, we identified 104 genes as ‘common ISGs’ (Figure 2) using various datasets (Table 1). Furthermore, these genes were closely associated with the DEGs by HIV-1 infection (Figure 3) and the evolutionarily conserved ISGs in mammals (Figure 4). Therefore, we consider that these 104 ‘common ISGs’ can be robustly upregulated in HIV-1 target cells by IFN-I stimulus.

Similar to the ISG datasets (Table 1), the conditions of HIV-1 infected datasets are also different (Table 2). Moreover, the viral strains and the time after infection were various and publicly unavailable. Nevertheless, our results suggested that HIV-1 infection induces the expression of ISGs (Figure 3B), which is consistent with previous reports (Sedaghat et al., 2008; Nakano et al., 2017; Yamada et al., 2018), and ‘common ISGs’ were particularly upregulated by HIV-1 infection (Figure 3C). Importantly, recent studies have suggested that the anti-HIV-1 effects mediated by ISGs are specific for viral strains (Krapp et al., 2016; OhAinle et al., 2018). For example, transmitted/founder viruses, which are the initial viruses transmitted to newly infected individuals and establish nascent infection, are more resistant to IFN-I than chronically controlled viruses (Parrish et al., 2013; Iyer et al., 2017). It was of particular interest that *RSAD2* (encoding Viperin), an ISG against a broad range of viruses (Chin and Cresswell, 2001; Wang et al., 2007; Gizzi et al., 2018), exhibits anti-HIV-1 activity in a strain-specific manner (Lim et al., 2012). This gene was a ‘common ISG’ (Figure 2A) that was upregulated by HIV-1 infection and was evolutionarily conserved ISGs in mammals (Figure 4). These findings suggest that certain HIV-1 strains have evolved to escape from the antiviral action triggered by this gene, although *RSAD2* was evolutionarily conserved ISGs in mammals and sophisticated to combat viral infections.

Although information on the DEGs by HIV-1 infection in MDMs would be of interest, these data have been publicly unavailable to date. Future studies using such datasets will lead to a deeper understanding of the difference in the gene expression patterns by HIV-1 infections among various cell types. Moreover, our recent study indicated that a basal expression level of *BST2* (encoding tetherin), a ‘common ISG’ defined in this study, is sufficient to exhibit anti-HIV-1 activity without IFN-I stimulus (Lee et al., 2018; Urata et al., 2018; Yamada et al., 2018). It should be carefully considered that the upregulation of ISGs by IFN-I stimulus is not necessarily essential for controlling a virus infection, and that certain genes, including ISGs, can potently inhibit HIV-1 replication in an IFN-I-independent manner.

In summary, these results suggest that our bioinformatic investigation provides robust information, which can be useful for understanding the relationship between the effect of IFN-I and the modulation of gene expression by HIV-1 infection in future investigations.

## Data Availability

Publicly available datasets were analyzed in this study. This data can be found here: www.ncbi.nlm.nih.gov/sra.

## Author Contributions

HA, JI, and KS conceived the study and designed the analyses. HA and JI performed bioinformatics analyses. KS wrote the manuscript. HA, JI, YK, and KS edited the manuscript. All authors read and approved the final manuscript.

## Conflict of Interest Statement

The authors declare that the research was conducted in the absence of any commercial or financial relationships that could be construed as a potential conflict of interest.

## Footnotes

1 www.ncbi.nlm.nih.gov/sra

2 www.ncbi.github.io/sra-tools

3 www.gencodegenes.org

4 www.ncbi.nlm.nih.gov:/genome/annotation_euk/Homo_sapiens/109
