# Supplementary material for: Comparative Description of the Expression Profile of Interferon-Stimulated Genes in Multiple Cell Lineages Targeted by HIV-1 Infection
Source: Front Microbiol. 2019 Mar 12;10:429. doi: 10.3389/fmicb.2019.00429 (PMC6423081; doi:10.3389/fmicb.2019.00429)
Supplement: Supplementary file 1 [file Data_Sheet_1.PDF]

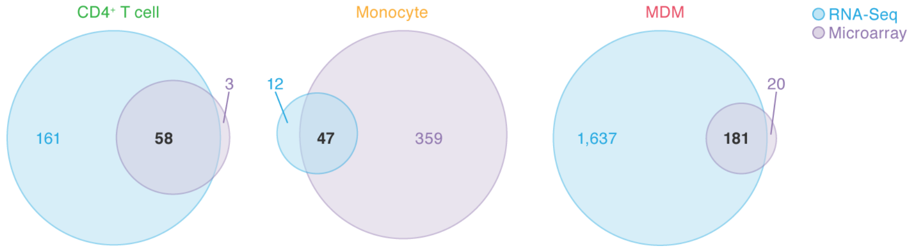

Supplementary Figure 1. Venn diagrams of the ISGs in CD4^{+} T cells, monocytes, and MDMs. The numbers of the ISGs identified by RNA-Seq (blue) and microarray (purple) in CD4^{+} T cells (left), monocytes (middle) and MDMs (right) are summarized.

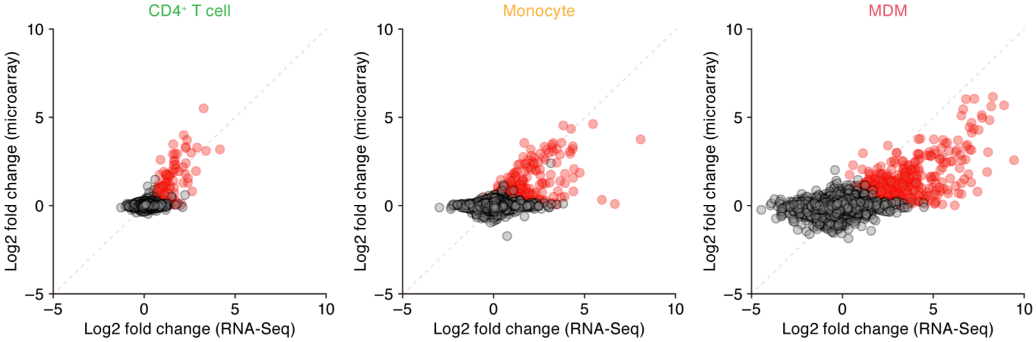

Supplementary Figure 2. A linear correlation of the induction levels of genes following IFN-I stimuli between RNA-Seq and microarray datasets. The log2 fold change values of gene expressions on RNA-Seq (x-axis) and microarray (y-axis) in CD4^{+} T cells (left), monocytes (middle) and MDMs (right) are summarized. Each dot indicates a gene, and the ISGs shown in **Figure 1A** and listed in **Table S5** are indicated in red.

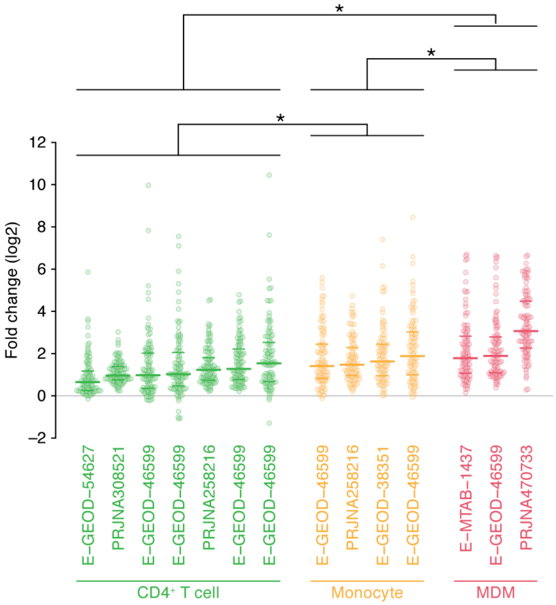

Supplementary Figure 3. Magnitude of the induction levels of 104 'common ISGs' in each dataset. Each dot indicates the fold change of an ISG expression by IFN-I treatment. Horizontal line indicates the quantiles. Asterisks indicate $P < 0.005$ by Welch's t test. The results from CD4^{+} T cells (7 datasets), monocytes (4 datasets) and MDMs (3 datasets) are shown. The summarized results are shown in **Figure 2C**.
